# Supplementary material for: Integrated exome and RNA sequencing of TFE3-translocation renal cell carcinoma
Source: Nat Commun. 2021 Sep 6;12:5262. doi: 10.1038/s41467-021-25618-z (PMC8421377; doi:10.1038/s41467-021-25618-z)
Supplement: Supplementary file 1 — Supplementary Information [file 41467_2021_25618_MOESM1_ESM.pdf]

Supplementary Figure 1

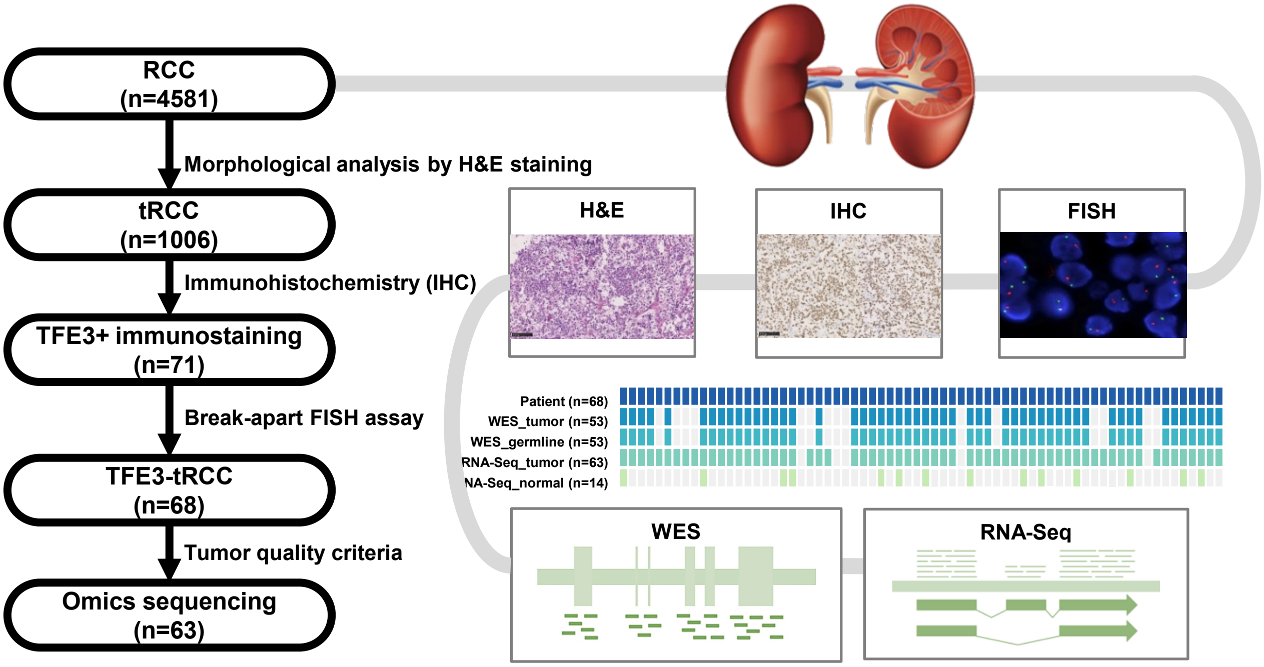

Supplementary Figure 1. CONSORT flow-diagram indicates screening process of *TFE3*-tRCC and disposition for the various analyses.

TFE3-tRCC = *TFE3*-translocation renal cell carcinoma.

Supplementary Figure 2

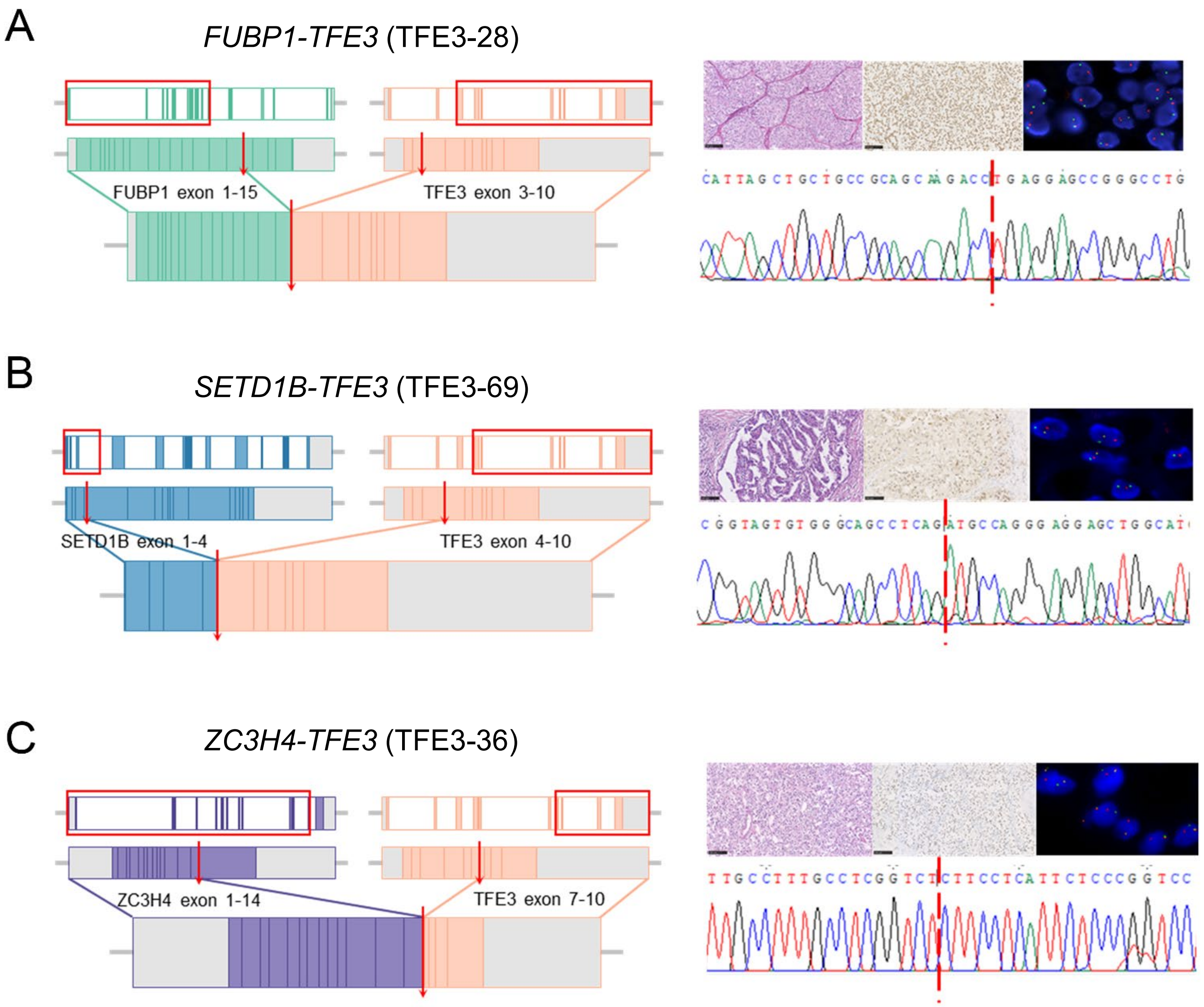

Supplementary Figure 2. Validation of three rare gene fusions.
Schematic of *FUBP1-TFE3* (A), *SETD1B-TFE3* (B) and *ZC3H4-TFE3* (C) fusion genes. The region in the red box includes the retained exons of fusion genes, and the red arrow denotes the breakpoints in the mRNA (upper panel). Pathologic images (H&E, TFE3 IHC and break-apart FISH assay, top) and sequencing chromatogram (bottom) are showed on the right. The red dashed line represents the breakpoint. Five random high-power fields were used for pathologic evaluation (H&E staining). Five random high-power fields were checked for TFE3 positive expression. One hundred non-overlapping tumor nuclei were evaluated for the rearrangement signals (break-apart FISH assay). IHC = immunohistochemistry. Magnification × 200. Scale bar = 100μm.

Supplementary Figure 3

A

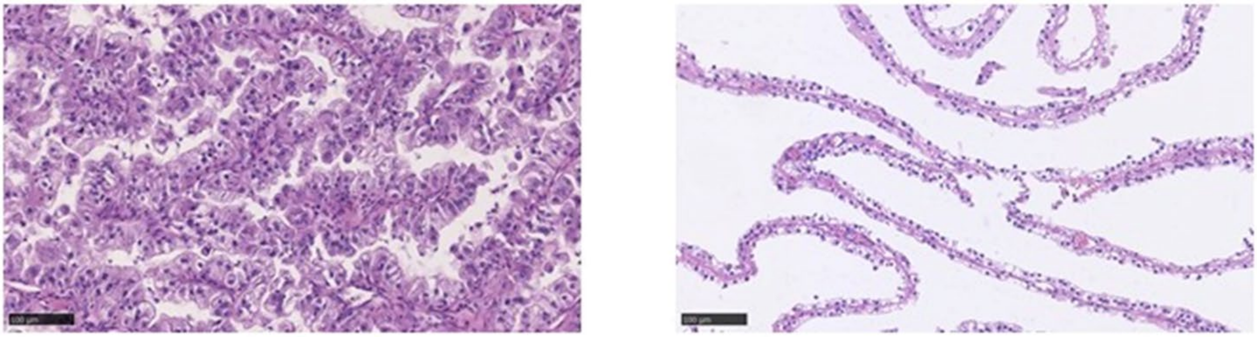

ASPSCR1-TFE3 (TFE3-32) *MED15-TFE3* (TFE3-21)

B

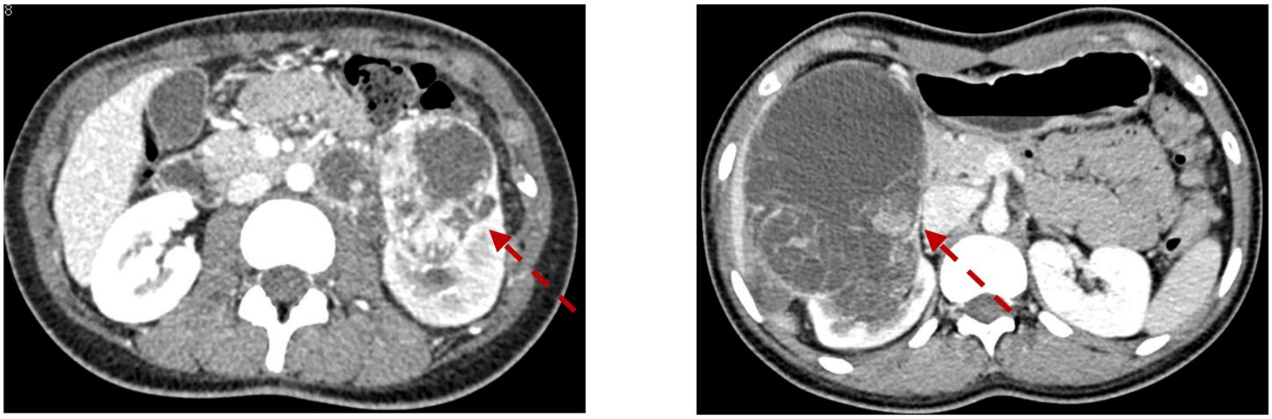

ASPSCR1-TFE3 (TFE3-24) *MED15-TFE3* (TFE3-38)

Supplementary Figure 3. Morphologic and Radiologic features of tumors with *ASPSCR1-TFE3* and *MED15-TFE3* fusion.

(A) Typical morphologic images of tumors with *ASPSCR1-TFE3* (left) and *MED15-TFE3* fusion (right). Five random high-power fields were used for pathologic evaluation (H&E staining). Magnification × 200. Scale bar = 100μm.

(B) Typical radiologic images in contrast-enhanced coronal reformat CT of tumors with *ASPSCR1-TFE3* (left) and *MED15-TFE3* fusion (right).

Supplementary Figure 4

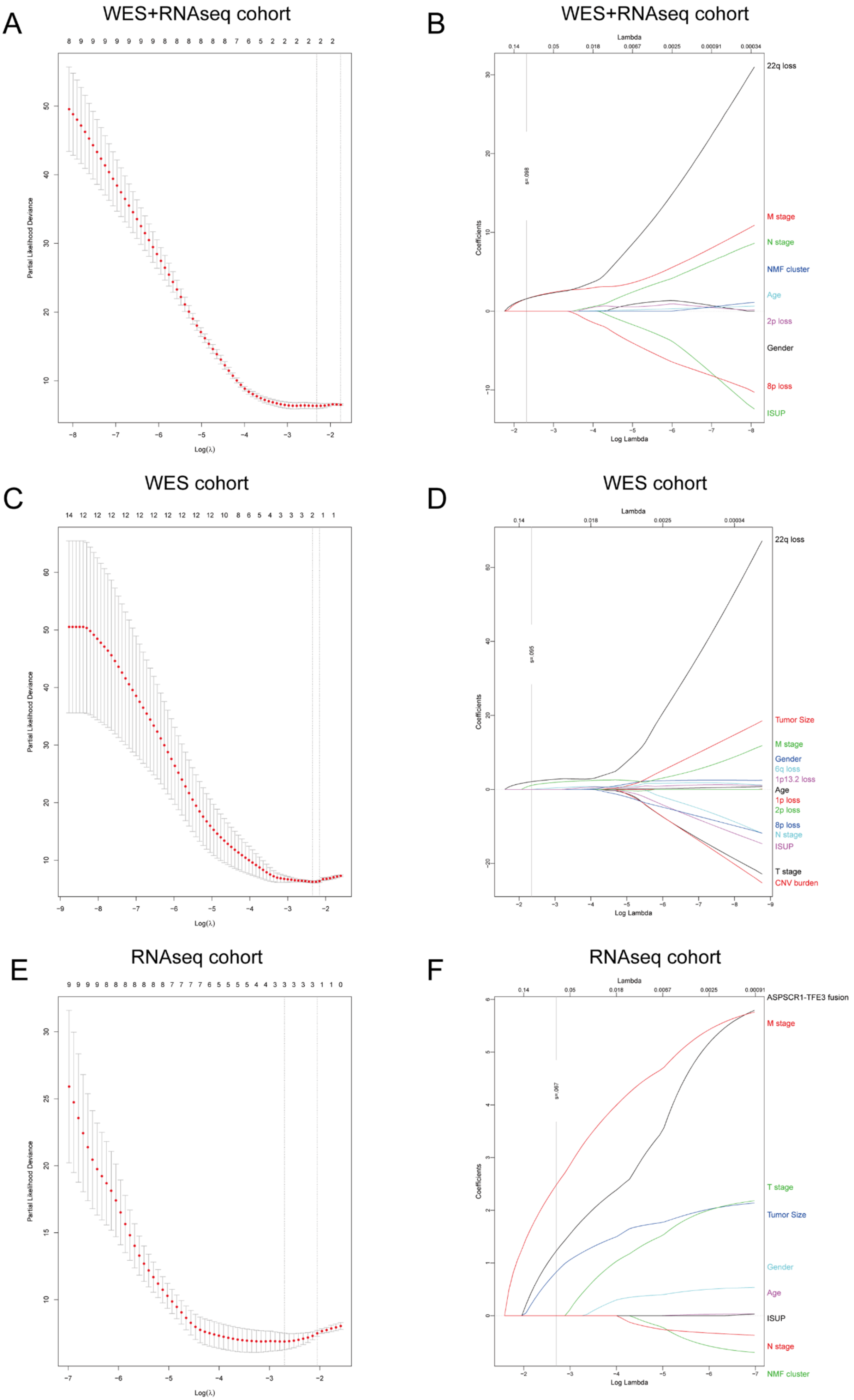

Supplementary Figure 4. Identify potential predictors for overall survival using LASSO cox regression.

(A) LASSO coefficient profiles of 16 prognosticators in WES+RNAseq cohort (n = 44).
(C) LASSO coefficient profiles of 14 prognosticators in WES cohort (n = 53).
(E) LASSO coefficient profiles of 9 prognosticators in WES cohort (n = 63).
(B, D and F) Cross-validation for turning parameter selection via minimum criteria in the LASSO regression model.

Data were expressed as mean ± SD (A, C, E). OS = overall survival, LASSO = least absolute shrinkage and selection operator, SD = standard deviation.

Supplementary Figure 5

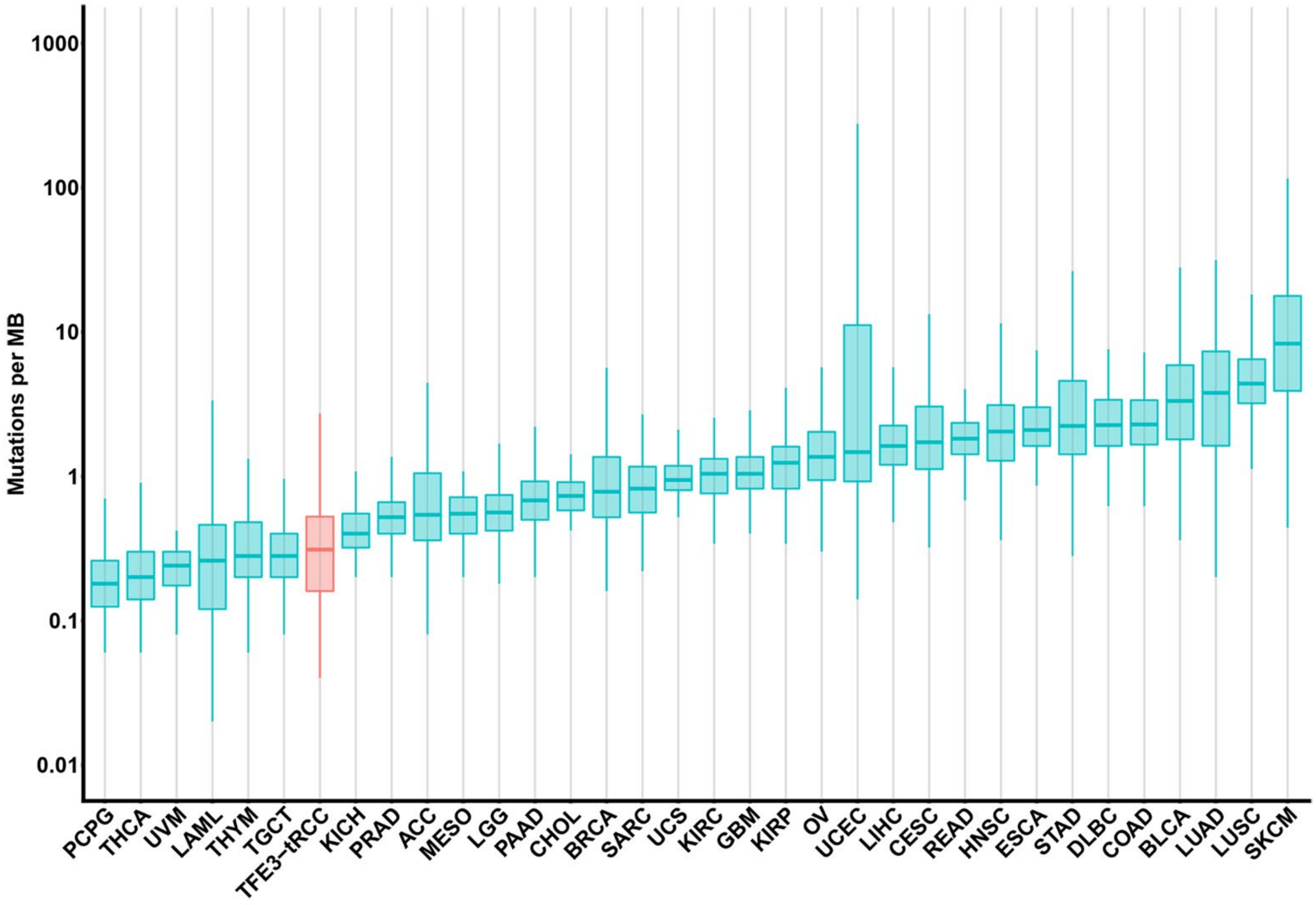

Supplementary Figure 5. Tumor mutation burden in *TFE3*-tRCC and compare with 33 tumor types from TCGA.

TFE3-tRCC = *TFE3*-translocation renal cell carcinoma (n = 53), ACC = adrenocortical carcinoma (n = 92), BLCA = bladder urothelial carcinoma (n = 411), BRCA = invasive breast carcinoma (n = 1020), CESC = cervical and endocervical cancers (n = 289), CHOL = cholangiocarcinoma (n = 36), COAD = colon adenocarcinoma (n = 404), DBLC = lymphoid neoplasm diffuse large B-cell lymphoma (n = 37), ESCA = esophageal carcinoma (n = 184), GMB = glioblastoma multiforme (n = 388), HNSC = head and neck squamous cell carcinoma (n = 507), KICH = chromophobe renal cell carcinoma (n = 66), KIRC = renal clear cell carcinoma (n = 369), KIRP = renal papillary cell carcinoma (n = 281), LAML = acute myeloid leukemia (n = 137), LGG = brain lower grade glioma (n = 510), LIHC = liver hepatocellular carcinoma (n = 363), LUAD = lung adenocarcinoma (n = 514), LUSC = lung squamous cell carcinoma (n = 458), MESO = mesothelioma (n = 82), OV = ovarian serous cystadenocarcinoma (n = 411), PAAD = pancreatic adenocarcinoma (n = 175), PCPG = pheochromocytoma and paraganglioma (n = 178), PRAD = prostate adenocarcinoma (n = 494), READ = rectum adenocarcinoma (n = 149), SARC = sarcoma (n = 236), SKCM = skin cutaneous melanoma (n = 466), STAD=stomach adenocarcinoma (n = 438), TGCT = testicular germ cell tumors (n = 128), THCA = thyroid carcinoma (n = 491), THYM = thymoma (n = 123), UCEC = uterine corpus endometrial carcinoma (n = 530), UCS=uterine carcinosarcoma (n = 57), UVM = uveal melanoma (n = 80).

Box plots show median levels (middle line), 25th and 75th percentile (box), and the whiskers. The upper whisker = min(max(x), Q3 + 1.5 × IQR), the lower whisker = max(min(x), Q1 − 1.5 × IQR). TCGA = The Cancer Genome Atlas, TMB = Tumor mutation burden, IQR = interquartile range.

Supplementary Figure 6

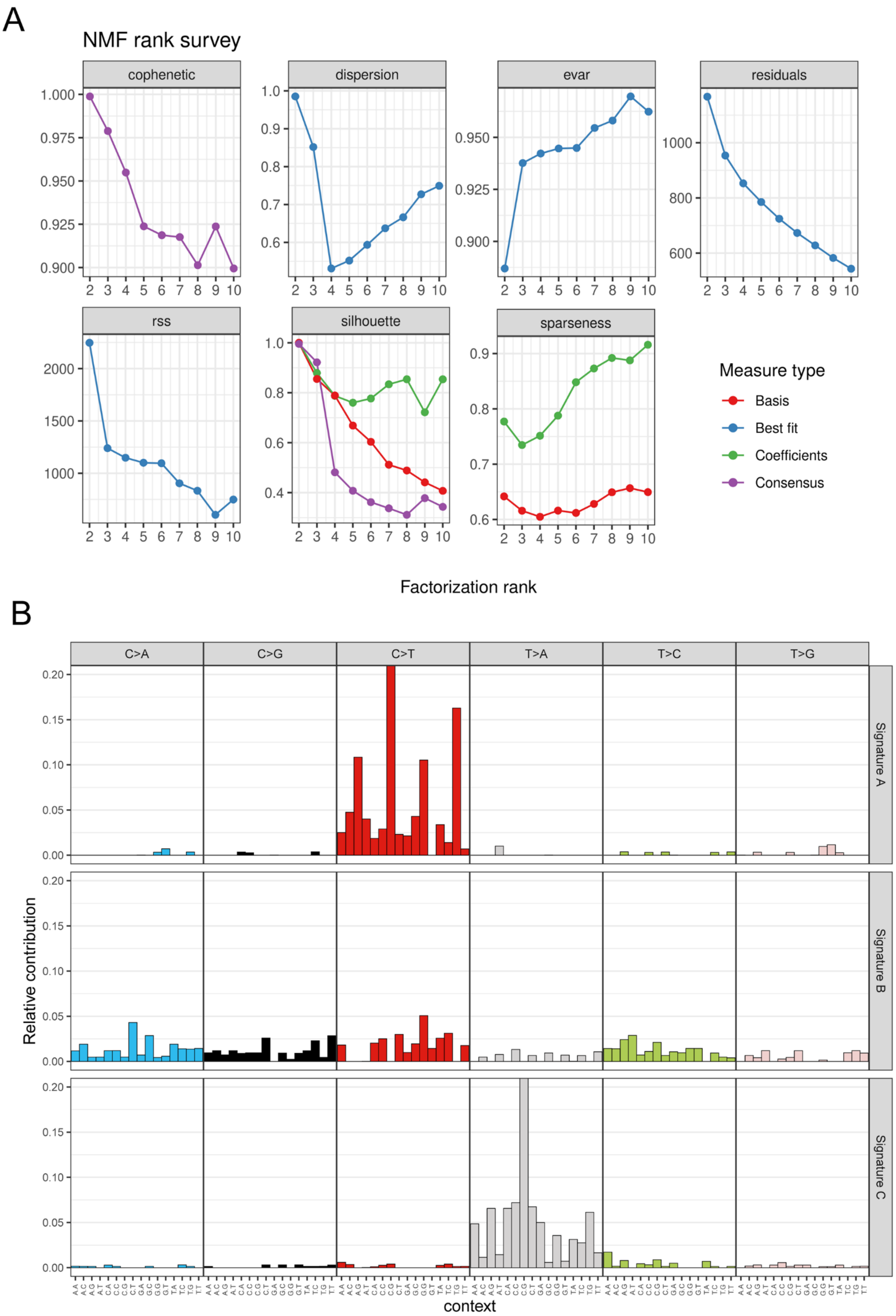

Supplementary Figure 6. Mutational signatures analysis.

(A) Metrics plot showing the optimal solution contained three signatures.

(B) Mutational signature barplots. Signature A corresponds to SBS87 and SBS1, Signature B corresponds to SBS40, and Signature C corresponds to SBS22. These corresponding signatures are defined by COSMIC mutational signatures v3.2 (https://cancer.sanger.ac.uk/cosmic/signatures).

SBS = single-base substitution.

Supplementary Figure 7

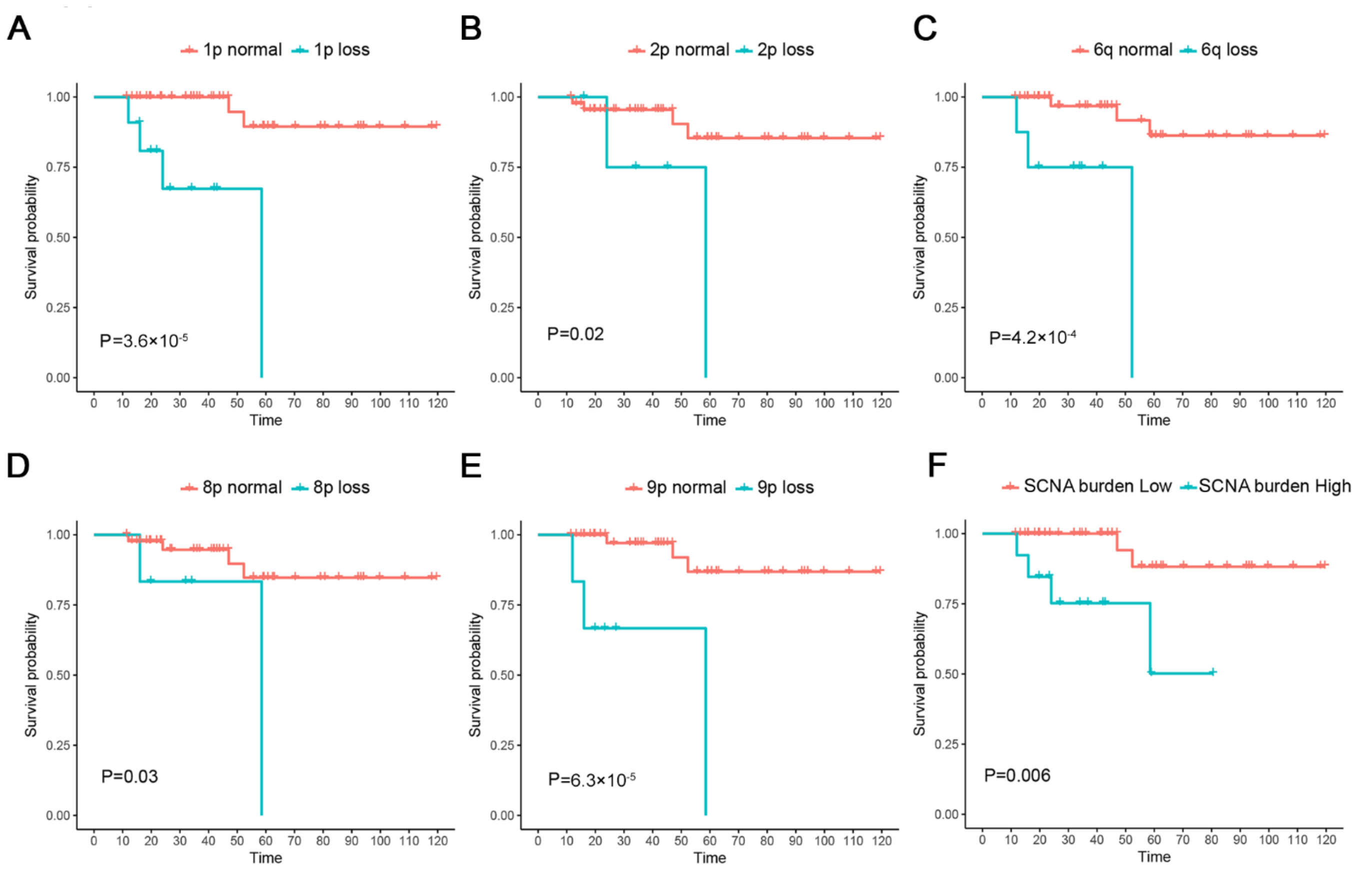

Supplementary Figure 7. SCNAs associated with overall survival.
(A-E) Overall survival by the status of 6 chromosome regions with loss.
(F) Overall survival by the status of SCNA burden. SCNA burden ≥ 75th percentile of SCNA burden was defined as SCNA high.
P-values were determined by two-sided log-rank test (A-F). SCNA = Somatic copy number alterations.

Supplementary Figure 8

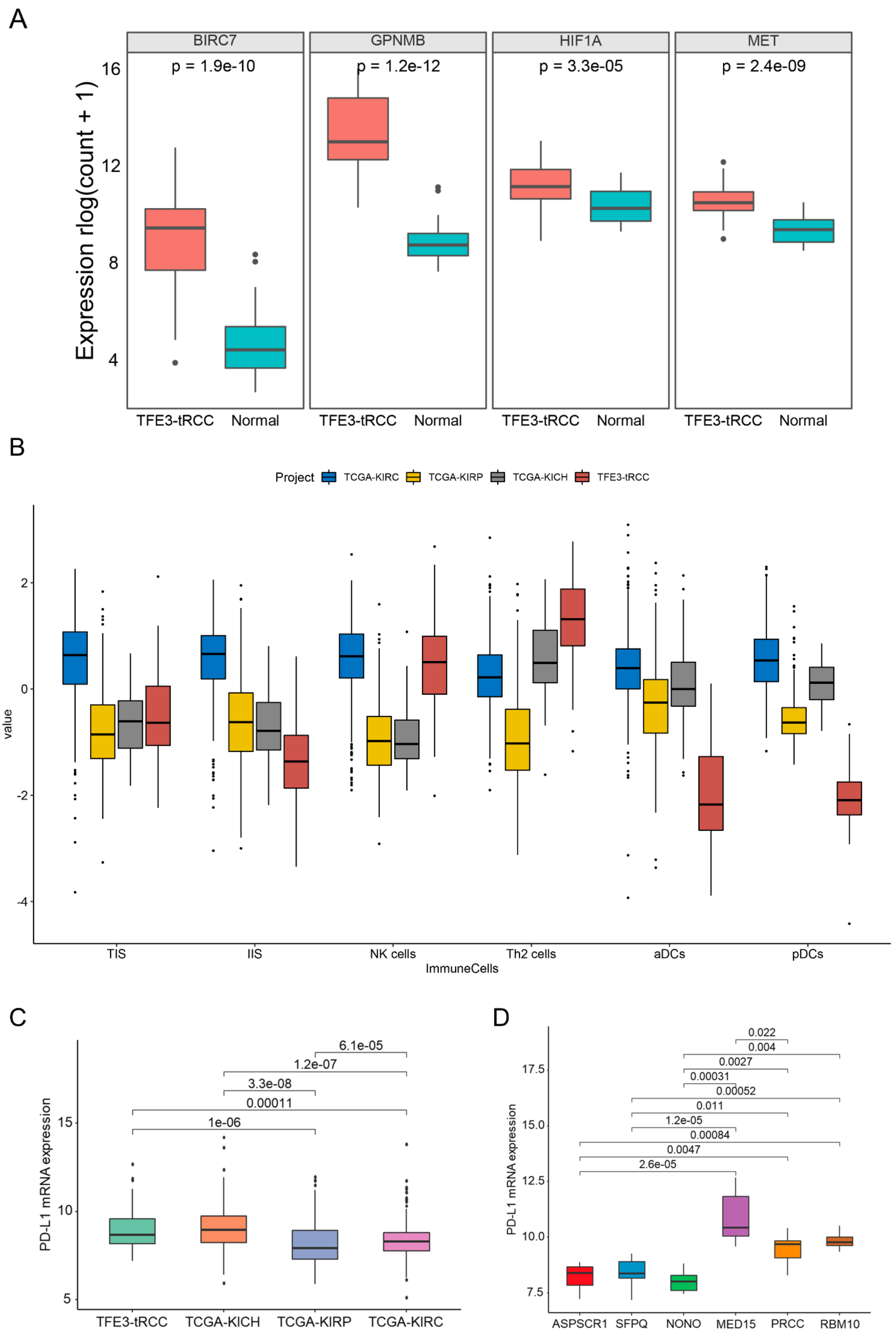

Supplementary Figure 8. Different mRNA expression in *TFE3*-tRCC

(A) Box plots show mRNA expression levels (counts) of indicated genes (*BIRC7*, *GPNMB*, *HIF1A* and *MET*) in tumors (red, n =63) and adjacent normal tissues (green, n = 14).

(B) Differential expression for each gene signature was analyzed between the TCGA-RCC and our *TFE3*-tRCC cohorts (n = 63). KICH (n = 65), KIRC (n = 539), KIRP (n = 289).

(C) Comparative expression of *PD-L1* mRNA expression between the different histological RCC. *TFE3*-tRCC (n = 63), KICH (n = 65), KIRC (n = 539), KIRP (n = 289).

(D) Comparative expression of *PD-L1* mRNA expression between the different *TFE3*-tRCC subtypes. *SFPQ-TFE3* (n=15), *ASPSCR1-TFE3* (n=13), *NONO-TFE3* (n=8), *MED15-TFE3* (n=8), *PRCC-TFE3* (n=6) and *RBM10-TFE3* fusions (n=4).

Box plots show median levels (middle line), 25th and 75th percentile (box), 1.5 times the interquartile range (whiskers) as well as outliers (single points). *P*-values were determined by the two-sides Mann-Whitney U test (A, C, D). *TFE3*-tRCC = *TFE3*-translocation renal cell carcinoma, TCGA = The Cancer Genome Atlas, KICH = chromophobe renal cell carcinoma, KIRC = renal clear cell carcinoma, KIRP = renal papillary cell carcinoma.

Supplementary Figure 9

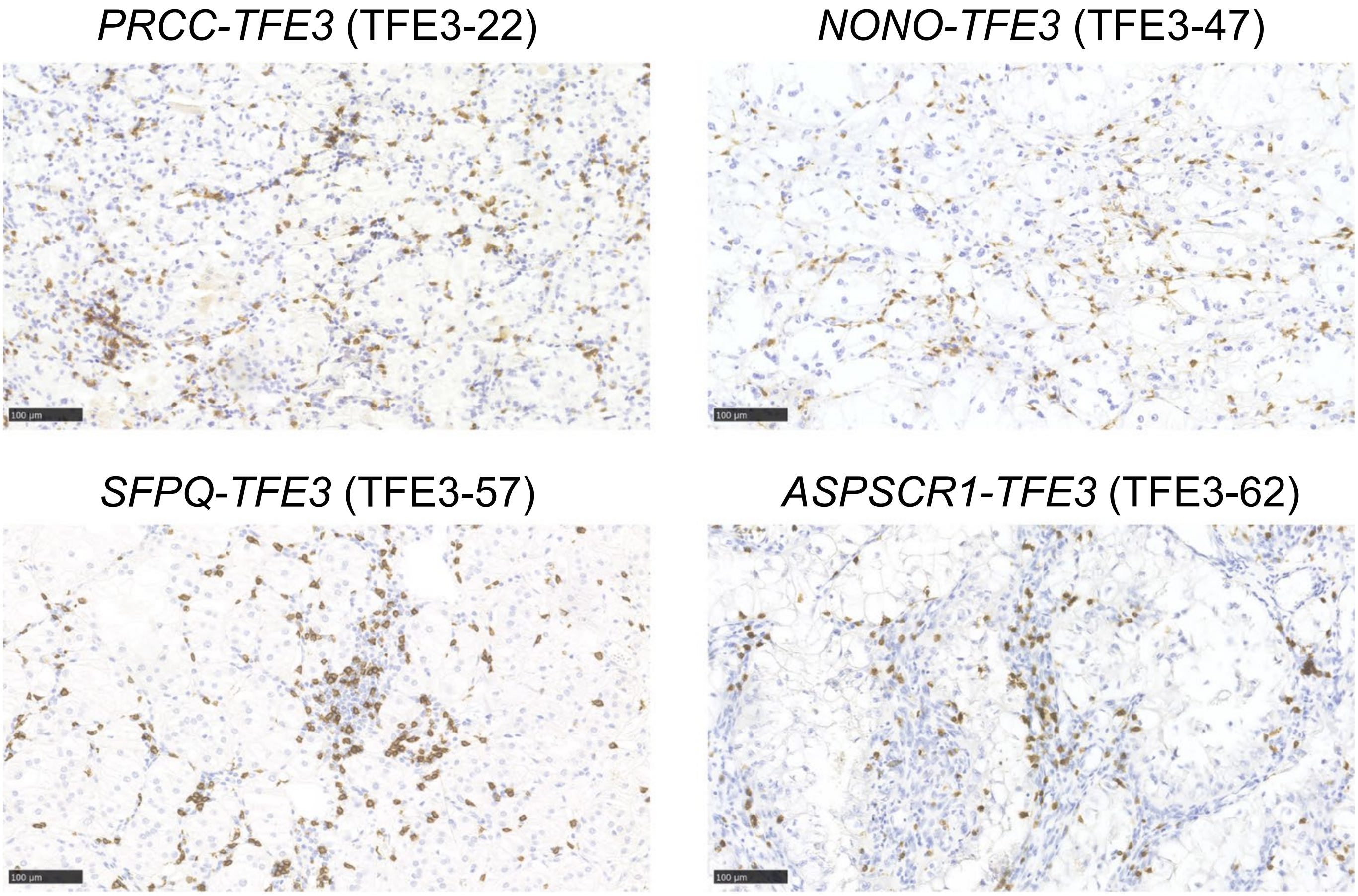

Supplementary Figure 9. CD8 expression in *TFE3*-tRCC.
Images of IHC staining of samples from patients with high CD8+ T cell infiltration in four selected samples (TFE3-22, TFE3-47, TFE3-57, and TFE3-62) from our cohort. Ten random high-power fields of tumor parenchyma were checked for CD8 positive expression. Magnification × 200. Scale bar = 100μm.
TFE3-tRCC = *TFE3*-translocation renal cell carcinoma.

Supplementary Figure 10

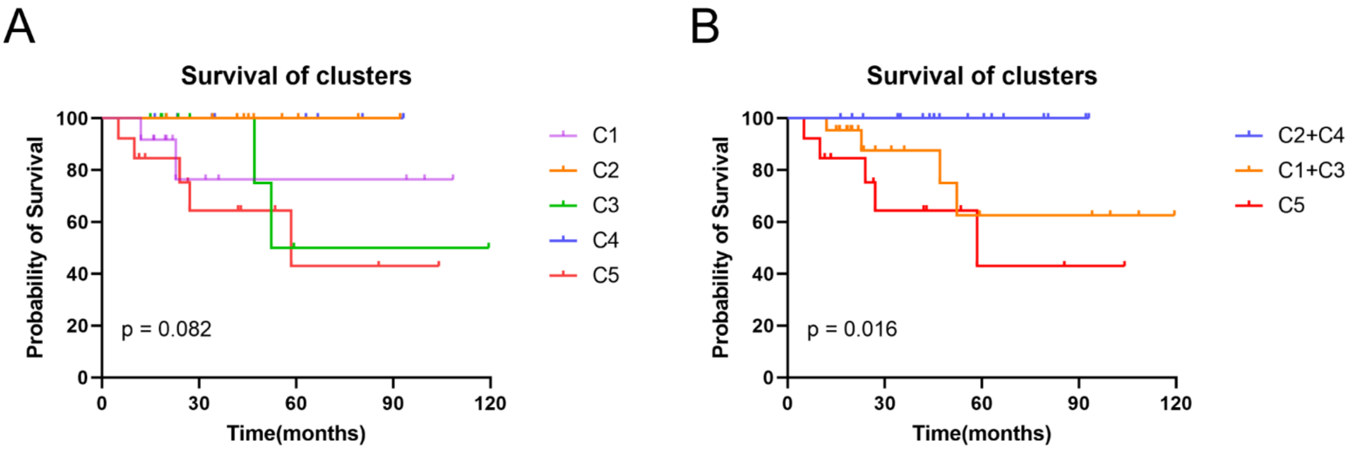

Supplementary Figure 10. Survival analysis of *TFE3*-tRCC NMF cluster

(A-B) Kaplan-Meier curves show the OS for patients with different NMF clusters. *P*-values were determined by two-sided log-rank test. C1, n = 12; C2, n = 11; C3, n = 10; C4, n = 8; C5, n = 13.

TFE3-tRCC = *TFE3*-translocation renal cell carcinoma, OS = overall survival, NMF = non-negative matrix factorization.

Supplementary Table 1. Baseline clinicopathologic characteristics of different *TFE3*-tRCC fusion subtypes.

Characteristics	Fusion Subtype							
	SFPQ-TFE3 **(n=15)**	***ASPSCR1-TFE3*** **(n=13)**	***NONO-TFE3*** **(n=8)**	***MED15-TFE3*** **(n=8)**	***PRCC-TFE3*** **(n=6)**	***RBM10-TFE3*** **(n=4)**	**Others (n=3)**	**Unknown (n=6)**
**Female, n (%)**	10 (66.7)	10 (76.9)	3 (37.5)	6 (75.0)	4 (66.7)	1 (25.0)	2 (66.7)	3 (50.0)
**Age, median (range)**	34 (12-69)	22 (6-52)	37.5 (18-70)	43 (20-70)	35 (24-42)	56 (37-63)	43 (42-56)	28 (10-58)
**Tumor Size, median (cm, range)**	4.7 (2.8-17.5)	6.0(2.0-19.6)	4.9 (2.4-19.4)	5.9 (3.3-13.4)	3.9 (1.4-6.1)	4.5 (3.8-10.0)	11.0 (6.5-11.2)	3.4 (3.0-5.5)
**T Stage, n (%)**								
≤ T2	15(100.0)	10 (76.9)	6 (75.0)	7 (87.5)	6(100.0)	2 (50.0)	2 (66.7)	5 (83.3)
≥ T3	0(0.0)	3 (23.1)	2 (25.0)	1 (12.5)	0(0.0)	2 (50.0)	1 (33.3)	1 (16.7)
**N stage, n (%)**								
N0	13 (86.7)	7 (53.8)	6 (75.0)	7 (87.5)	5(83.3)	4 (100.0)	2 (66.7)	4 (66.7)
N1	2 (13.3)	6 (46.2)	2 (25.0)	1 (12.5)	1(16.7)	0(0.0)	1 (33.3)	2 (33.3)
**M, n (%)**								
M0	13 (86.7)	12 (92.3)	6(75.0)	8(100.0)	5 (83.3)	4 (100.0)	1 (33.3)	6 (100.0)
M1	2 (13.3)	1 (7.7)	2(25.0)	0(0.0)	1 (16.7)	0(0.0)	2 (66.7)	0 (0.0)
**ISUP grade, n (%)**								
≤ 2	6 (40.0)	2 (15.4)	7(87.5)	8(100.0)	4(66.7)	0(0.0)	1 (33.3)	1 (16.7)
≥ 3	9 (60.0)	11 (84.6)	1(12.5)	0(0.0)	2(33.3)	4 (100.0)	2 (66.7)	5 (83.3)

ISUP = The International Society of Urological Pathology.

P-values were determined by Pearson's chi-square test.

Supplementary Table 2. Multivariate analyses of clinicopathologic and genomic features in predicting OS

	WES+RNA-seq cohort (n=44, variates=16)		WES cohort (n=53, variates=14)		RNA-seq cohort (n=63, variates=9)	
	HR (95%CI)	*P*-Value	HR (95%CI)	*P*-Value	HR (95%CI)	*P*-Value
**Tumor size, cm**<br>≥ Median vs. < Median	-	-	-	-	7.88 (1.24-50.22)	0.029
**M stage**<br>1 vs. 0	35.16 (2.58-479.06)	0.008	32.79 (2.58-416.11)	0.007	93.08 (7.44-1164.81)	4.37×10^{-4}
**22p loss**<br>Yes vs. No	30.32 (3.00-306.6)	0.004	52.49 (5.58-493.64)	0.001	-	-
***ASPSCR1-TFE3* fusion**<br>Yes vs. No	-	-	-	-	17.42 (2.00-151.72)	0.010

WES+RNA-seq cohort: Adjusted for Gender, Age, Tumor size, ISUP grade, T stage, N stage, M stage, SCNA burden, 1p loss, 2p loss, 6q loss, 8p loss, 9p loss, 22p loss, *ASPSCR1-TFE3* fusion and NMF cluster.

WES cohort: Adjusted for Gender, Age, Tumor size, ISUP grade, T stage, N stage, M stage, SCNA burden, 1p loss, 2p loss, 6q loss, 8p loss, 9p loss and 22p loss.

RNA-seq cohort: Adjusted for Gender, Age, Tumor size, ISUP grade, T stage, N stage, M stage, *ASPSCR1-TFE3* fusion, NMF cluster.

Covariate *P*-values derived from z-scores are two-sided.

OS = overall survival, WES = whole-exome sequencing, SCNA = somatic copy number alteration, ISUP = The International Society of Urological Pathology, NMF = non-negative matrix factorization, HR = hazard ratio, CI = confidence interval.

Supplementary Table 3. Univariate survival analysis of frequent mutation genes and somatic copy number alterations

Covariate	Levels	Number	Median OS (months)	2-year OS	Log-rank P
DST mutation	Yes vs. No	5 vs. 46	82.2 vs. 104.1	100% vs. 92.2%	0.842
DNAH8 mutation	Yes vs. No	3 vs. 48	-	100% vs. 92.7%	0.661
HMHA1 mutation	Yes vs. No	4 vs. 47	73.8 vs. 103.8	75.0% vs. 94.8%	0.442
TMB*	High vs. Low	12 vs. 39	110.9 vs. 100.1	91.7% vs. 93.6%	0.701
1p loss	Yes vs. No	11 vs. 40	45.4 vs. 112.1	66.2% vs. 100%	3.6×10^{-5}
2p loss	Yes vs. No	5 vs. 46	49.9 vs. 107.7	75.0% vs. 95.4%	0.018
6q loss	Yes vs. No	8 vs. 43	42.7 vs. 109.4	75.0% vs. 96.7%	4.2×10^{-4}
8p loss	Yes vs. No	6 vs. 45	51.4 vs. 107.2	83.3% vs. 94.5%	0.031
9p loss	Yes vs. No	6 vs. 45	43.7 vs. 109.6	66.7% vs. 97.0%	6.3×10^{-5}
22p loss	Yes vs. No	7 vs. 44	36.0 vs. 112.1	44.4% vs. 100%	7.9×10^{-8}
SCNA burden**	High vs. Low	13 vs. 38	59.5 vs. 111.3	74.7% vs. 100%	0.006

TMB = tumor mutation burden, SCNA = somatic copy number alteration.

* TMB ≥ 75th percentile of TMB was defined as TMB high, ** SCNA ≥ 75th percentile of SCNA was defined as SCNA high.

P-value was determined by two-sided log-rank test.

Supplementary Table 4. Baseline clinicopathologic characteristics of different somatic copy number alterations.

Characteristics	1p loss (n=11)	2p loss (n=5)	6q loss (n= 8)	8p loss (n=6)	9p loss (n=6)	22q loss (n=7)
**Age, median (range)**	25 (22-70)	39 (22-70)	31 (22-70)	31 (22-70)	31 (22-69)	24 (22-38)
**Gender, n (%)**						
Male	3 (27.3)	2 (40.0)	4 (50.0)	4 (66.7)	2 (33.3)	2 (28.6)
Female	8 (72.7)	3 (60.0)	4 (50.0)	2 (33.3)	4 (67.7)	5 (71.4)
**Tumor size, median (cm, range)**	5.6 (2.1-17.5)	6.5 (3.3-6.9)	5.6 (4.0-17.5)	5.6 (4.9-13.4)	6.2 (5.0-17.5)	5.6 (2.0-17.5)
**T stage, n (%)**						
≤T2	11 (100.0)	5 (100.0)	7 (87.5)	6 (100.0)	5 (83.3)	6 (85.7)
≥T3	0 (0.0)	0 (0.0)	1 (12.5)	0 (0.0)	1 (16.7)	1 (14.3)
**N stage, n (%)**						
N0	7 (63.6)	3 (60.0)	6 (75.0)	4 (66.7)	2 (33.3)	2 (28.6)
N1	4 (36.4)	2 (40.0)	2 (25.0)	2 (33.3)	4 (67.7)	5 (71.4)
**M stage, n (%)**						
M0	10 (90.9)	5 (100.0)	6 (75.0)	6 (100.0)	5 (83.3)	6 (85.7)
M1	1 (9.1)	0 (0.0)	2 (25.0)	0 (0.0)	1 (16.7)	1 (14.3)
**ISUP grade, n (%)**						
≤ 2	5 (45.5)	3 (60.0)	3 (37.5)	2 (33.3)	1 (16.7)	0 (0.0)
≥ 3	6 (54.5)	2 (40.0)	5 (62.5)	4 (66.7)	5 (83.3)	7 (100.0)
***ASPSCR1-TFE3* fusion (n=8)**						
No	5 (50.0)	3 (60.0)	6 (85.7)	4 (80.0)	4 (80.0)	2 (33.3)
Yes	5 (50.0)	2 (40.0)	1 (14.3)	1 (20.0)	1 (20.0)	4 (66.7)
**TMB, n (%)**						
High (≥ 75th percentile)	1 (9.1)	0 (0.0)	2 (25.0)	2 (33.3)	3 (50.0)	1 (14.3)
Low (< 75th percentile)	10 (90.9)	5 (100.0)	6 (75.0)	4 (66.7)	3 (50.0)	6 (85.7)
**SCNA burden, n (%)**						
High (≥ 75th percentile)	8 (72.7)	3 (60.0)	5 (62.5)	4 (66.7)	6 (100.0)	6 (85.7)
Low (<75th percentile)	3 (27.3)	2 (40.0)	3 (37.5)	2 (33.3)	0 (0.0)	1 (14.3)

ISUP = The International Society of Urological Pathology, TMB = tumor mutation burden, SCNA = somatic copy number alteration.
P-values were determined by Pearson's chi-square test.

Supplementary Table 5. Primer sequences for fusion validation

Primer	Sequence
ZC3H4 exon 14	5’- CGGTGTCCCTGACTTCCTG-3’
TFE3 exon 7	5’-GCCTTTGCCTCGGTCT-3’
FUBP1 exon 15	5’-ACCAGGCCCGGCTCCTCA-3’
TFE3 exon 3	5’-GCCTGTTCCCGACGCTC-3’
SETD1B exon 4	5’- GCGTGGGGCGAGTCTCAT-3’
TFE3 exon 4	5’- GGACCCGATGGTGAGCAGC-3’
