# Supplementary material for: Integrated exome and RNA sequencing of TFE3-translocation renal cell carcinoma
Source: Nat Commun. 2021 Sep 6;12:5262. doi: 10.1038/s41467-021-25618-z (PMC8421377; doi:10.1038/s41467-021-25618-z)
Supplement: Supplementary file 3 — Description of Additional Supplementary Files [file 41467_2021_25618_MOESM3_ESM.pdf]

Description of Additional Supplementary Files

File Name: Supplementary Data 1
Description: Overview of patients and samples

File Name: Supplementary Data 2
Description: Exons and functional domains of the TFE3 and fusion partner genes

File Name: Supplementary Data 3
Description: Somatic mutations detected by WES

File Name: Supplementary Data 4
Description: Recurrent copy number alterations identified by FACETS and GISTIC analysis

File Name: Supplementary Data 5
Description: Differentially expressed genes by DESeq2 analysis

File Name: Supplementary Data 6
Description: Non-negative matrix factorization (NMF) clustering by QuSAGE analysis
