# Supplementary material for: Integrated exome and RNA sequencing of TFE3-translocation renal cell carcinoma
Source: Nat Commun. 2021 Sep 6;12:5262. doi: 10.1038/s41467-021-25618-z (PMC8421377; doi:10.1038/s41467-021-25618-z)
Supplement: Supplementary file 10 — Reporting Summary [file 41467_2021_25618_MOESM10_ESM.pdf]

nature portfolio

Corresponding author(s): Zhenhua Liu, Pengfei Shen, Ni Chen and Hao Zeng

Last updated by author(s): Jul 23, 2021

Reporting Summary

Nature Portfolio wishes to improve the reproducibility of the work that we publish. This form provides structure for consistency and transparency in reporting. For further information on Nature Portfolio policies, see our Editorial Policies and the Editorial Policy Checklist.

Statistics

For all statistical analyses, confirm that the following items are present in the figure legend, table legend, main text, or Methods section.

n/a	Confirmed	
☐	☒	The exact sample size (*n*) for each experimental group/condition, given as a discrete number and unit of measurement
☐	☒	A statement on whether measurements were taken from distinct samples or whether the same sample was measured repeatedly
☐	☒	The statistical test(s) used AND whether they are one- or two-sided<br>*Only common tests should be described solely by name; describe more complex techniques in the Methods section.*
☒	☐	A description of all covariates tested
☐	☒	A description of any assumptions or corrections, such as tests of normality and adjustment for multiple comparisons
☐	☒	A full description of the statistical parameters including central tendency (e.g. means) or other basic estimates (e.g. regression coefficient) AND variation (e.g. standard deviation) or associated estimates of uncertainty (e.g. confidence intervals)
☐	☒	For null hypothesis testing, the test statistic (e.g. *F*, *t*, *r*) with confidence intervals, effect sizes, degrees of freedom and *P* value noted<br>*Give P values as exact values whenever suitable.*
☒	☐	For Bayesian analysis, information on the choice of priors and Markov chain Monte Carlo settings
☒	☐	For hierarchical and complex designs, identification of the appropriate level for tests and full reporting of outcomes
☒	☐	Estimates of effect sizes (e.g. Cohen's *d*, Pearson's *r*), indicating how they were calculated

Our web collection on statistics for biologists contains articles on many of the points above.

Software and code

Policy information about availability of computer code

Data collection	No special software was used.
Data analysis	Here we listed the versions of all the software we used and all the details can be found in the Methods section. All softwares used in this study are publicly available and see the methods section for details.<br>STAR v2.7.0f;<br>STAR-Fusion v1.9.1;<br>NMF algorithm;<br>MEM algorithm;<br>BWA V0.7.17;<br>Sambamba version 0.6.8;<br>GATK v3.8;<br>MuTect2 v4.1.1.0;<br>MutationalPatterns v3.0.1;<br>FACETS v0.5.14;<br>GISTIC2 v2.0.23;<br>TSGene v2.0<br>R software v3.6.0;<br>SPSS v16.0.

For manuscripts utilizing custom algorithms or software that are central to the research but not yet described in published literature, software must be made available to editors and reviewers. We strongly encourage code deposition in a community repository (e.g. GitHub). See the Nature Portfolio guidelines for submitting code & software for further information.

Data

Policy information about availability of data

All manuscripts must include a data availability statement. This statement should provide the following information, where applicable:

- Accession codes, unique identifiers, or web links for publicly available datasets
- A description of any restrictions on data availability
- For clinical datasets or third party data, please ensure that the statement adheres to our policy

The WES data generated in this study have been deposited in NODE (The National Omics Data Encyclopedia) database (https://www.biosino.org/node/project/detail/OEP002535, accession numbers OEP002535) and the NCBI Sequence Read Archive (SRA) database (https://www.ncbi.nlm.nih.gov/bioproject/PRJNA701236, accession numbers PRJNA701236). The gene expression data reported in this paper were deposited in the Gene Expression Omnibus (GEO) database (https://www.ncbi.nlm.nih.gov/geo/query/acc.cgi?acc=GSE167573, accession numbers GSE167573). The transcriptome data of TCGA KIRC, KIRP and KICH were collected from the following web-links https://portal.gdc.cancer.gov/projects/TCGA-KIRC, https://portal.gdc.cancer.gov/projects/TCGA-KIRP and https://portal.gdc.cancer.gov/projects/TCGA-KICH, respectively. The remaining data are available within the Supplementary Information.

Field-specific reporting

Please select the one below that is the best fit for your research. If you are not sure, read the appropriate sections before making your selection.

☒ Life sciences ☐ Behavioural & social sciences ☐ Ecological, evolutionary & environmental sciences

For a reference copy of the document with all sections, see nature.com/documents/nr-reporting-summary-flat.pdf

Life sciences study design

All studies must disclose on these points even when the disclosure is negative.

Sample size	No statistical methods were used to determine the sample size. As TFE3-translocation renal cell carcinoma is a rare subtype of renal cell carcinoma, the sample size was determined by the access and availability of patient samples and materials.
Data exclusions	No data were excluded from the analyses
Replication	The detected FUBP1-TFE3, SETD1B-TFE3 and ZC3H4-TFE3 fusions by RNA sequencing were validated by PCR-Sanger sequencing.
Randomization	This is not relevant since we did not use different experimental groups or conditions in our study.
Blinding	This is not relevant since we did not use different experimental groups or conditions in our study.

Reporting for specific materials, systems and methods

We require information from authors about some types of materials, experimental systems and methods used in many studies. Here, indicate whether each material, system or method listed is relevant to your study. If you are not sure if a list item applies to your research, read the appropriate section before selecting a response.

Materials & experimental systems

n/a	Involved in the study	
☐	☒	Antibodies
☒	☐	Eukaryotic cell lines
☒	☐	Palaeontology and archaeology
☒	☐	Animals and other organisms
☐	☒	Human research participants
☒	☐	Clinical data
☒	☐	Dual use research of concern

Methods

n/a	Involved in the study	
☒	☐	ChIP-seq
☒	☐	Flow cytometry
☒	☐	MRI-based neuroimaging

Antibodies

Antibodies used	Anti-TFE3; clone: MRQ-37; dilution: 1:100; host species: rabbit; application: immunocytochemistry; MXB biotechnologies; cat# RMA-0063<br>Anti-PDL1; clone: 22C3; dilution: 1:50; host species: mouse; application: immunocytochemistry; Dako, Agilent; cat# M3653<br>Anti-CD8; clone: C8/144B; dilution: ready to use; host species: mouse; application: immunocytochemistry; Dako, Agilent; cat# IR623<br>Anti-HIF1A; clone: NA; dilution: 1:5000; host species: rabbit; application: immunocytochemistry; Novus Biologicals; cat# NB100-479<br>Anti-CD31; clone: UMAB30; dilution: ready to use; host species: mouse; application: immunocytochemistry; ZSGB-BIO; cat# ZM-0044

	Anti-CD8; clone: C8/144B; dilution: 1:200; host species: mouse; application: immunofluorescence; Cell Signaling; cat#CST70306<br>Anti-CD56; clone: 123C3; dilution: 1:400; host species: mouse; application: immunofluorescence; Cell Signaling; cat#CST3576<br>Anti-CD68; clone: BP6036; dilution: 1:100; host species: rabbit; application: immunofluorescence; BioLynx; cat#BX50031<br>Anti-HLA-DR; clone: EPR3692; dilution: 1:250; host species: rabbit; application: immunofluorescence; Abcam; cat#ab92511<br>Anti-panCK; clone: PAN-CK (Cocktail); dilution: 1ug/ml; host species: mouse; application: immunofluorescence; Abcam; cat#ab215838<br>Anti-S100; clone: NA; dilution: 1:100; host species: rabbit; application: immunofluorescence; Gene Tech; cat#GZ031129<br>DAPI; dilution: 1:100; application: immunofluorescence; Sigma-Aldrich; Cat#D9542<br>PANO 7-plex IHC kit (TSA-RM); Panovue; cat#0004100100
Validation	We have provided a link for the relevant data sheet for each antibody. The data sheet includes the manufacturer's validations statements, quality control procedures and relevant citations:<br>Anti-TFE3; http://www.maxim.com.cn/sitecn/xpsd/7325.html<br>Anti-PDL1; https://www.agilent.com.cn/cs/library/packageinsert/public/D40230%20M365329-8CN%20RUO%20IFU.pdf<br>Anti-CD8 (immunocytochemistry); https://www.agilent.com.cn/zh-cn/product/clinical-flow-cytometry/reagents-for-clinical-flow-cytometry/clinical-single-color-antibodies/cd8-787412#productdetails<br>Anti-HIF1A; https://www.novusbio.com/products/hif-1-alpha-antibody_nb100-479<br>Anti-CD31; http://www.zsbio.com/product/ZM-0044<br>Anti-CD8 (immunofluorescence); https://www.cellsignal.cn/products/primary-antibodies/cd8a-c8-144b-mouse-mab-ihc-specific/70306?site-search-type=Products&N=4294956287&Ntt=cd8&fromPage=plp<br>Anti-CD56; https://www.cellsignal.cn/products/primary-antibodies/ncam1-cd56-123c3-mouse-mab/3576?site-search-type=Products&N=4294956287&Ntt=123c3&fromPage=plp&_requestid=75837<br>Anti-CD68; http://www.biolynx.cn/products/methods/112/138/303.html<br>Anti-HLA-DR; https://www.abcam.cn/hla-dr-antibody-epr3692-ab92511.html<br>Anti-panCK; https://www.abcam.cn/pan-cytokeratin-antibody-pan-ck-cocktail-ab215838.html<br>Anti-S100; https://www.genetech.com.cn/goods/goods_detail/469.html<br>DAPI; https://www.sigmaaldrich.cn/CN/zh/product/sigma/d9542?context=product<br>PANO 7-plex IHC kit (TSA-RM); http://www.panovue.cn/news/417338401973473280

Human research participants

Policy information about studies involving human research participants

Population characteristics	The median age at diagnosis was 32.5 years (range: 5-70 years) and 9 (13.2%) patients were younger than 18 years old. The male: female ratio was 2:3. The median tumor size was 4.7 cm (range: 1.4-19.6 cm). At initial diagnosis, 16 (23.5%) and 7 (10.3%) patients presented with regional lymph node metastasis and distant metastasis, respectively. Morphologically, TFE3-tRCCs presented with diverse architectural and cytologic features, including papillary, tubular, acinar and cystic patterns, and 37 (54.4%) tumors had a ISUP grade ≥ 3. For primary kidney tumors, 27 (39.7%) and 41 (60.3%) patients underwent nephron-sparing surgery and radical nephrectomy, respectively, and 10 (14.7%) died at the end of follow-up (median 43.8 months, 95% CI: 31.5-56.1). All relevant information concerning this population used in this study is included in Table 1 and S1.
Recruitment	A total of 4,581 cases diagnosed as RCC who underwent surgery for the kidney at our center between 2009 and 2019 were reviewed by two experienced uropathologists (Ni Chen and Mengni Zhang). Among them, 1,006 suspicious non-clear cell RCC cases were identified via morphological evaluation and were selected for further TFE3 immunohistochemistry (IHC). As a result, 68 TFE3 positive cases were confirmed as TFE3-tRCC by break-apart FISH assay (Figure S1). Patients were selected only if they had no prior treatment before surgery and if the tumor samples available. Because of the rarity of TFE3-tRCC, we enrolled as many patients as possible in this study. Therefore, no self-selection bias was present.
Ethics oversight	The study was conducted in accordance with the Declaration of Helsinki and was approved by the Ethics Committee of West China Hospital of Sichuan University. All patients or family members provided written consent for genetic analysis.

Note that full information on the approval of the study protocol must also be provided in the manuscript.
